# Importance of the Posterior Plate in Three‐Column Tibial Plateau Fractures: A Finite Element Analysis and Clinical Validation

**DOI:** 10.1111/os.14021

**Published:** 2024-03-04

**Authors:** Chen‐dong Liu, Sun‐jun Hu, Shi‐Min Chang, Shou‐chao Du, Wen‐feng Xiong, Yong‐qian Chu

**Affiliations:** ^1^ Department of Orthopedic Surgery Yangpu Hospital, School of Medicine, Tongji University Shanghai China

**Keywords:** dual plates, posterior column, three columns, tibial plateau fractures, triple plates

## Abstract

**Objective:**

Dual‐plate fixation was thought to be the gold standard for treating complicated bicondylar tibial plateau fractures, yet it was found to be hard to accommodate the posterior column in three‐column fractures. Currently, column‐specific fixation is becoming more and more recognized, but no comprehensive investigation has been performed to back it up. Therefore, the objective of this study was to validate the importance of posterior column fixation in the three‐column tibial fractures by a finite element (FE) analysis and clinical study.

**Methods:**

In FE analysis, three models were developed: the longitudinal triple‐plate group (LTPG), the oblique triple‐plate group (OTPG), and the dual‐plate group (DPG). Three loading scenarios were simulated. The distribution of the displacement and the equivalent von Mises stress (VMS) in each structure was calculated. The comparative measurements including the maximum posterior column collapse (MPCC), the maximum total displacement of the model (MTD), the maximum VMS of cortical posterior column (MPC‐VMS), and the maximum VMS located on each group of plates and screws (MPS‐VMS). The clinical study evaluated the indicators between the groups with or without the posterior plate, including operation time, blood loss volume, full‐weight bearing period, Hospital for Special Surgery Knee Scoring system (HSS), Rasmussen score, and common postoperative complications.

**Results:**

In the FE analysis, the MPCC, the MPC‐VMS, and the MTD were detected in much lower amounts in LTPG and OTPG than in DPG. In comparison with DPG, the LTPG and OTPG had larger MPS‐VMS. In the clinical study, 35 cases were included. In the triple‐plate (14) and dual‐plate (21) groups, the operation took 115.6 min and 100.5 min (*p* < 0.05), respectively. Blood loss in both groups was 287.0 mL and 206.6 mL (*p* < 0.05), and the full‐weight bearing period was 14.5 weeks and 16.2 weeks (*p* < 0.05). At the final follow‐up, the HSS score was 85.0 in the triple‐plate group and 77.5 in the dual‐plate (*p* < 0.05), the Rasmussen score was 24.1 and 21.6 (*p* < 0.05), there were two cases with reduction loss (9.5%) in the dual‐plate group and one case of superficial incision infection found in the triple‐plate group.

**Conclusion:**

The posterior implant was beneficial in optimizing the biomechanical stability and functional outcomes in the three‐column tibial plateau fractures.

## Graphical abstract

In this work, we performed an FE analysis and a clinical study to verify that it was important to employ a posterior implant in maintaining the anatomical reduction and reducing the risk of secondary articular collapse which were associated with a better functional outcome in the three‐column tibial plateau fractures.

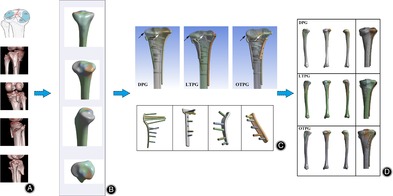

## Introduction

Tibial plateau fractures rarely occur and account for approximately 9.2% of all tibial fractures.^1^ Complex tibial plateau fractures frequently happen due to high‐energy violence, such as car or bicycle accidents. They are often accompanied by severe comminution, substantial soft tissue damage, or neurovascular injury.^2^, ^3^ With the development of the classification of tibial plateau fractures, different surgical protocols for various types of fractures emerged. In 1979, Schatzker et al.^4^ introduced the X‐ray‐based Schatzker classification which focused on the medial and lateral column and categorized the bicondylar fractures into V or VI type, identifying the medial and lateral fragments well in the anteroposterior view, but it was not available in visualizing the posterior fragment. In 2010, Luo et al.^5^ introduced the “Three‐column” classification through the computed tomography (CT) technology. They further classified the tibial plateau into the medial, lateral, and posterior column, illustrating that the fractures involving the three columns were prone to failure and that supporting the posterior column by a posterior plate was necessary.

A supplemental implant on the posterior column in three‐column fractures is highly advised because the posterior column involvement has been identified as a passive prognostic factor associated with poor functional outcomes, such as pain, knee‐related quality of life, and activities of daily living.^6^, ^7^ Controversially, it has been generally documented that the dual‐plate method for medial and lateral columns is enough to maintain anatomical reduction and enable early rehabilitation for the majority of bicondylar fractures in previous studies.^8^, ^9^, ^10^ Nevertheless, previous study demonstrated that 43% of fractures involved the posteromedial fragment and the majority of posteromedial fragments exhibited a mediolateral fracture line orientation (PMAFA <68°) and that may predispose them to insufficient stabilization with anterolateral implant.^11^ As a result, some authors have advocated the triple‐plate method, even the multiple‐plate method, emphasizing the importance of the specific stabilization of each column.^5^, ^11^, ^12^, ^13^

In 2014, Chang et al.^14^ proposed the “bicondylar four‐quadrant” concept, demonstrating a complex three‐column fracture type and further subdividing the posterior column into the posteromedial and the posterolateral sub‐columns. They noted the posteromedial column typically had a V‐shape or L‐shape spike, and two posterior sub‐columns tended to displacement and clarified that the medial and lateral columns could be easily restored by stabilizing two displaced posterior sub‐columns in advance. They obtained a satisfactory outcome by offering a column‐specific fixation mode of L‐shaped locking plate for anterolateral, and multiple reconstruction plates for posteromedial, posterolateral, and anteromedial fragments.

However, the aforementioned viewpoints were presented from clinical studies rather than rigorous experimental validation. It is our contention that the posterior implant plays a critical role in maintaining anatomical reduction by covering the posterior spike in three‐column fractures, which directly impacts postoperative function. Additionally, another implant enhances stability of the posterior column, facilitating early rehabilitation. Oblique fixation and longitudinal fixation are the most common methods of placement in posterior column fractures.^5^, ^14^ Oblique placement offers broader coverage and is commonly used for fractures in the posterolateral column, while longitudinal placement is frequently employed for posteromedial column fractures due to the physiological stresses endured by this column. Nevertheless, the biomechanical distinctions between the two placements in fractures involving both the posteromedial and posterolateral columns remain unknown. Building upon the above considerations, the objectives of this study can be summarized as follows: (i) to investigate whether there are biomechanical and clinical benefits associated with utilizing a posterior plate in three‐column fractures through finite element (FE) analysis and retrospective clinical study, and (ii) to explore the optimal biomechanical placement of the posterior plate.

## Materials and Methods

### Finite Element Study

#### FE Fracture Model

**FIGURE 1:**
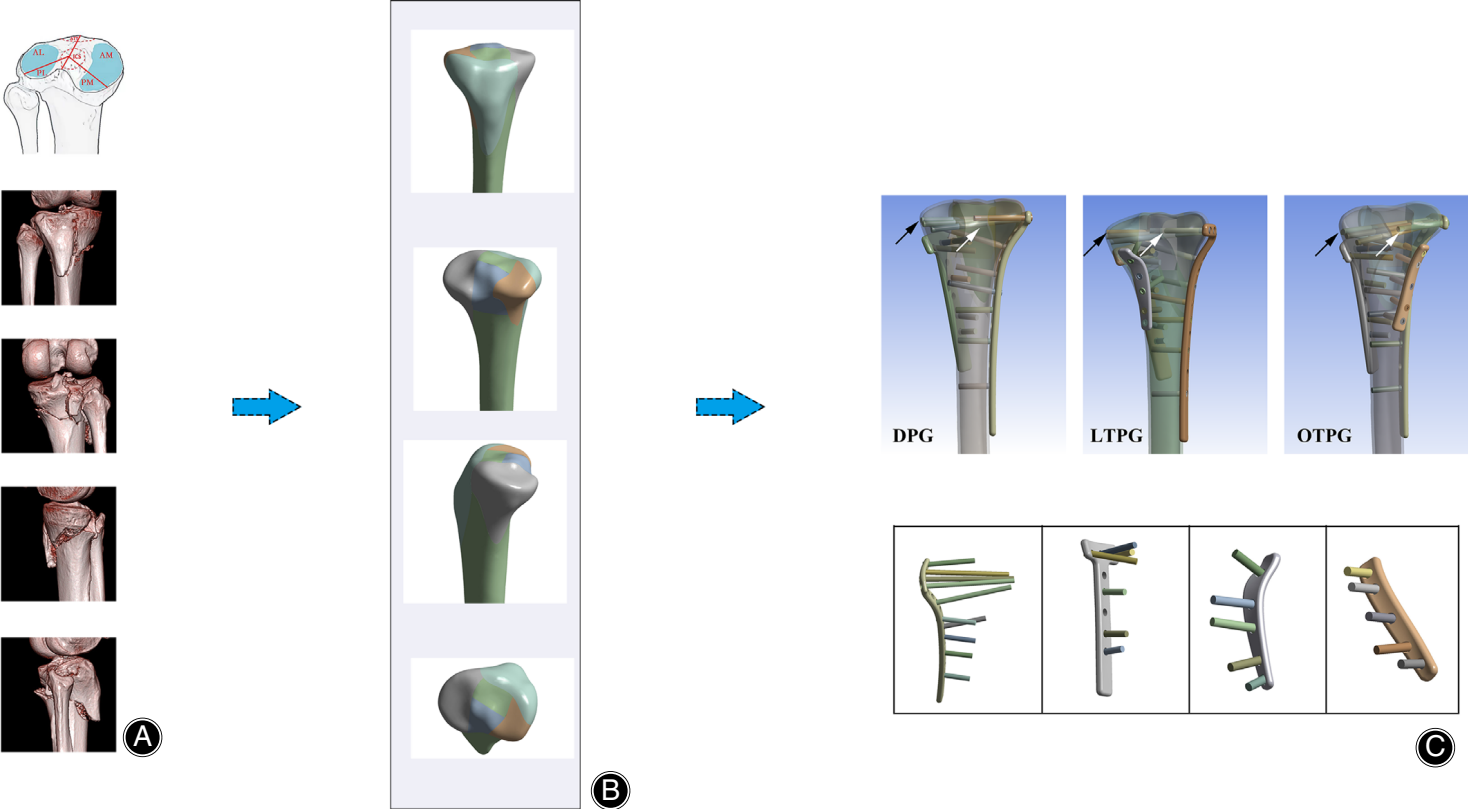
The procedure for building the FE models. (A) The presentation of the “bicondylar four‐quadrant theory” and the corresponding patient's CT data. (B) The tibial plateau fracture model's segmentation in the different views. (C) The black arrows pointed to the bi‐cortical mode, and the white arrows demonstrated that the proximal screws of L‐shaped plates were all through the posterior column fragment at approximately 90°. The inferior image exhibited the contoured implants and the distribution of the constructs.

A 42‐year‐old healthy male (height: 172 cm, weight: 63 kg, BMI: 21.3 kg/m², with no history of osteoporosis, osteoarthritis, or fractures) underwent CT scanning using a 64‐slice CT scanner (Siemens AG, Erlangen, Germany) to acquire data with a slice thickness of 1 mm. The resulting data were saved in DICOM format and imported into Mimics software (version 21.0, Materialize, Leuven, Belgium). The geometry data of the cortical and the cancellous bone was extracted within the “Region grow” module according to the exclusive gray values of tissues with different densities on the CT image. The authors initially processed the image, including filling the hole and smoothing the surface before exporting the STL file. The STL file was imported into Geomagic Wrap (version 2017, 3D Systems, Rock Hill, SC, USA) for smoothing, denoising, and local optimization. The thickness of cortical bone was set to be 2 mm artificially.^15^ A complex three‐column fracture type (bicondylar four‐quadrant) was produced using a typical patient's clinical CT data. This bicondylar four‐quadrant fracture type corresponded to the schematic drawing of the “bicondylar four‐quadrant classification” and previous tibial plateau fractures mapping.^14^, ^16^, ^17^ The fracture was modeled on a CT‐derived 3D model of the knee with an intact tibial plateau in Solidworks (version 2017, Dassault Systèmes, Waltham, MA, USA) (Figure 1A,B).

#### FE Experimental Models

The models of plates and screws were created by the parameter from the manufacturer (Synthes, Oberdorf, Switzerland) and then were implemented into the assembly. Three assembly models were ultimately established and were divided into the following groups: longitudinal triple‐plate group (LTPG): anterolateral L‐shaped anatomical locking plate, medial T‐shaped plate for the proximal tibia, and posteromedial reconstruction plate in longitudinal fixation; oblique triple‐plate group (OTPG): anterolateral L‐shaped anatomical locking plate, medial T‐shaped plate for the proximal tibia, posterolateral reconstruction plate in oblique fixation; dual plates group (DPG): anterolateral L‐shaped anatomical locking plate, and the medial T‐shaped plate for the proximal tibia. According to the injury mechanism and the fracture morphology of the established model, the primary L‐shaped plate was put on the anterolateral side to buttress the collapsed articular surface, the T‐shaped plate and the reconstruction plate were put on the medial side and posterior side for the supporting effect.^18^ All screws of the L‐shaped plate were set in bi‐cortical mode, and the proximal screws were all designed to penetrate through the posterior column perpendicularly for playing the most substantial property.^17^ The screws in the two other plates were set in uni‐cortical mode (Figure 1C).

**FIGURE 2:**
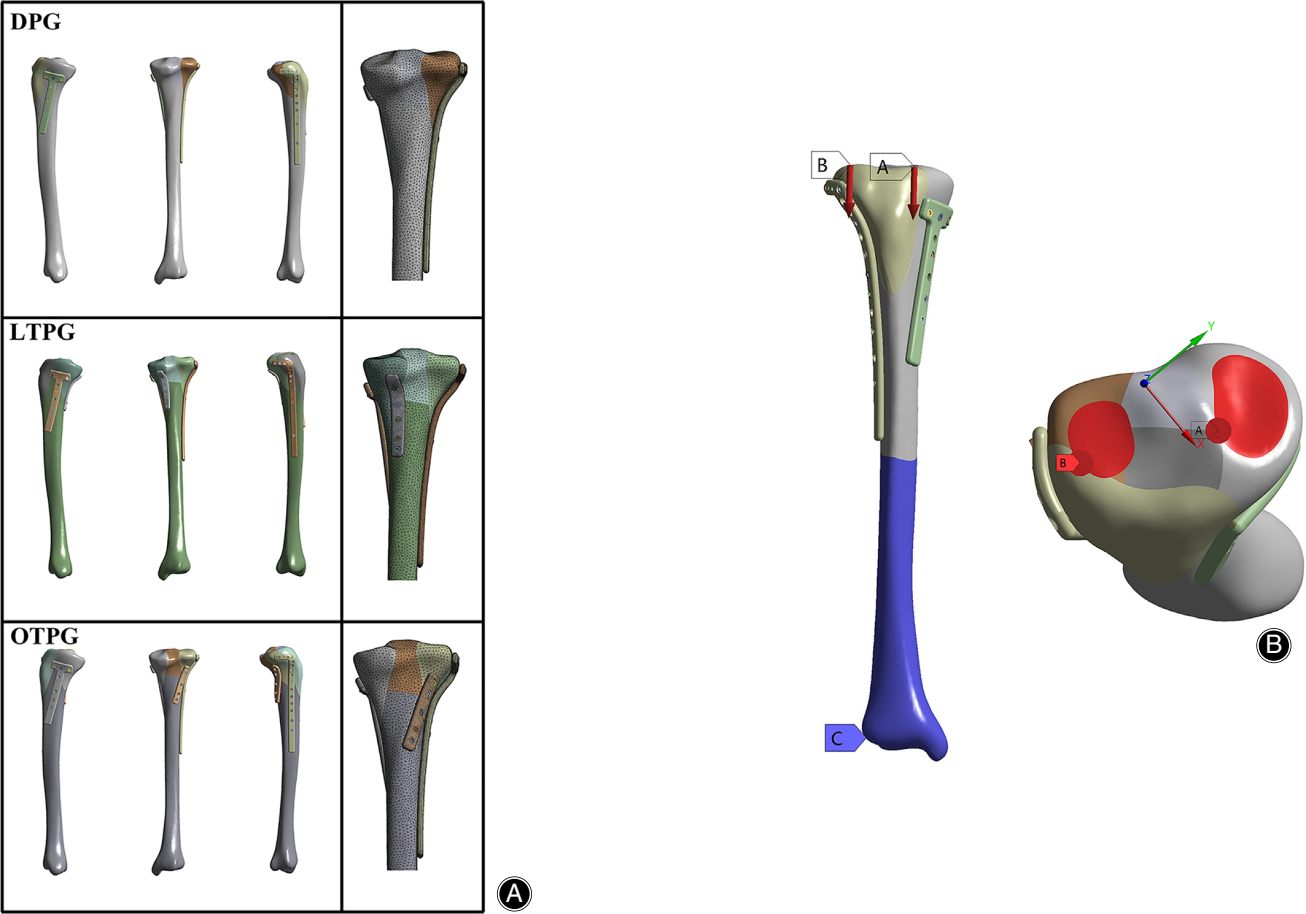
(A) FE assemblies of three groups and the corresponding meshing image. (B) The constraint condition and regional loading distribution of the FE model. The distal end of the tibial was fixed (blue area), and the two different forces was set axially (two red arrows). The axial force was distributed over the medial column (label A) and lateral column (label B) at 6:4.

According to previous studies,^19^, ^20^, ^21^, ^22^ the material properties of the plates, screws, and bone were assumed to be linearly elastic, isotropic, and homogenous. Young's modulus and Poisson's ratio was performed in Table 1. Finally, finite element mesh processing was done in Ansys workbench (version 2022, Ansys, Canonsburg, PA, USA). To improve the analysis accuracy and obtain a great operational convergence, a total of five meshing tests were conducted to attain an adaptive value of element and node number with a deviation of stress less than 5%, and the strain was below 1%.^23^, ^24^ The resulting mesh was the 10‐node quadratic tetrahedral element with a size of 2.5 mm of bone and 1 mm of plates and screws. The number of elements were 345,782, 376,266, and 378,924 in the assembly of the DGP, LTPG, OTPG, and the nodes were 557,864, 607,693, and 611,177, respectively (Figure 2A).

**TABLE 1:** Material assignment of the FE models.

Components	Young's modulus (MPa)	Poisson's ratio
Cortical bone	15,000	0.3
Cancellous bone	100	0.3
Titanium	110,000	0.3

#### Loading and Boundary Conditions

The distal end of the model was completely constrained when an axial load of 600, 1200, and 1800N (the ultimate load in human gait was roughly three times the body weight) was applied along the mechanical axis, simulating the discontinuous physiological scenarios of a 60 kg adult during gait.^25^ Sixty per cent of the load was distributed over the medial plateau, while 40% was distributed over the lateral.^15^, ^26^ The boundary conditions were established based on previous literature^22^, ^27^, ^28^: (1) the contacts between the fragments, between the cortical bone and the screws, between the cancellous bone and the screws were modeled with a friction coefficient of 0.4, 0.8, and 0.8, respectively; (2) the friction coefficient of the contact surface between the plates and cortical bone was 0.3; (3) the plate and screws were fully bonded without friction to simulate the locking‐compressing effect (Figure 2B).

#### Evaluation Parameters

The parameters for each construct included the distribution and the maximum value of the von Mises stress (VMS) and the displacement. Maximum posterior column collapse (MPCC), which was defined as the axial maximum displacement of the cancellous posterior column fragment and considered the primary indicator of the stability of the tibial plateau, was also chosen to assess the degree of the articular subsidence of the posterior column.^29^ To compare the overall stability, the maximum total displacement of the model (MTD) was taken into account. The maximum VMS of the cortical posterior column (MPC‐VMS) was chosen to depict the peak stress concentration area. In order to pinpoint the weak area of the implants and assess their resistance, the maximum VMS on plates and screws (MPS‐VMS) was recorded. The computerized calculation results were performed by GraphPad Prism (version 9.5, GraphPad Software, Boston, MA, USA).

### Clinical Study

#### Demographics

From January 2016 to January 2022, 42 cases of three‐column tibial plateau fractures were enrolled, and seven cases were excluded due to loss of follow‐up or insufficient data. A total of 35 cases with an average age of 47 were included in our case series. An anterolateral combined with a posteromedial approach was adopted. The triple‐plate method (medial, lateral, and posterior plate) was used in 14 of the cases (nine of OTPG and five of LTPG), while the dual‐plate method (medial and lateral) was used in the other cases. The posterior plate served as a “position implant,” which could maintain the posterior reduction for ease of the main plate placement during the surgery and also enhanced the posterior fragment to avoid the secondary movement postoperatively. Therefore, we chose the oblique fixation for those with unstable posterolateral columns and longitudinal fixation for the unstable posteromedial columns, which placement was determined to be employed depending on which sub‐column has been involved and the oblique fixation tended to be more utilized if both sub‐columns were broken. The baseline data is exhibited in Table 2. All patients were followed up for at least 12 months (12–25 months). This study was approved by the institutional review board of Yangpu Hospital, Tongji University School of Medicine (LL‐2018‐ZRKX‐024).

**TABLE 2:** Demographics.

	Triple‐plate group (14)	Dual‐plate group (21)	*p*‐value
Gender			>0.05
Male	12	18	
Female	2	3	
Affected side			>0.05
Left	8	9	
Right	6	12	
Schatzker classification			>0.05
V	12	18	
VI	2	3	
AO/OTA classification			>0.05
C1	8	15	
C2	2	1	
C3	4	5	
Injury cause			>0.05
Traffic accidents	18	10	
Falls	3	4	

#### Postoperative Management and Outcome Evaluation

The operation time and the blood loss volumes of all patients were recorded. The follow‐up period and full‐weight bearing period were documented throughout the successive follow‐up. The full‐weight bearing period was calculated as the amount of time since the patient was able to walk normally without the use of any assistive devices, depending on whether the X‐rays showed that the trabecular bone had crossed the gap and the fracture line had disappeared. The Hospital for Special Surgery score and the Rasmussen score^30^ were utilized for functional measurement at the final follow‐up. In addition, the common postoperative complications, including implant failure, vascular injury, peroneal nerve palsy, thrombosis, fracture delayed healing, articular collapse, and superficial incision infection, were examined at each follow‐up. All patients had a similar rehabilitation protocol. After the surgery, a continuous passive motion machine was employed in the hospital for 3 days at least. Beginning in the fourth to eighth postoperative week, partial weight‐bearing was encouraged. Full weight‐bearing was postponed until the fracture had healed and a callus surfaced on X‐ray.

### Statistical Analysis

Statistical data analysis was compared by SPSS (version 22.0, IBM, Armonk, NY, USA). Continuous and categorical data were analyzed using the *t*‐test and chi‐squared test, respectively. The value of *p* <0.05 was considered statistically significant. The outcome data were presented as mean ± standard deviation (SD).

## Results

### The Regional Stability of the Posterior Column

**FIGURE 3:**
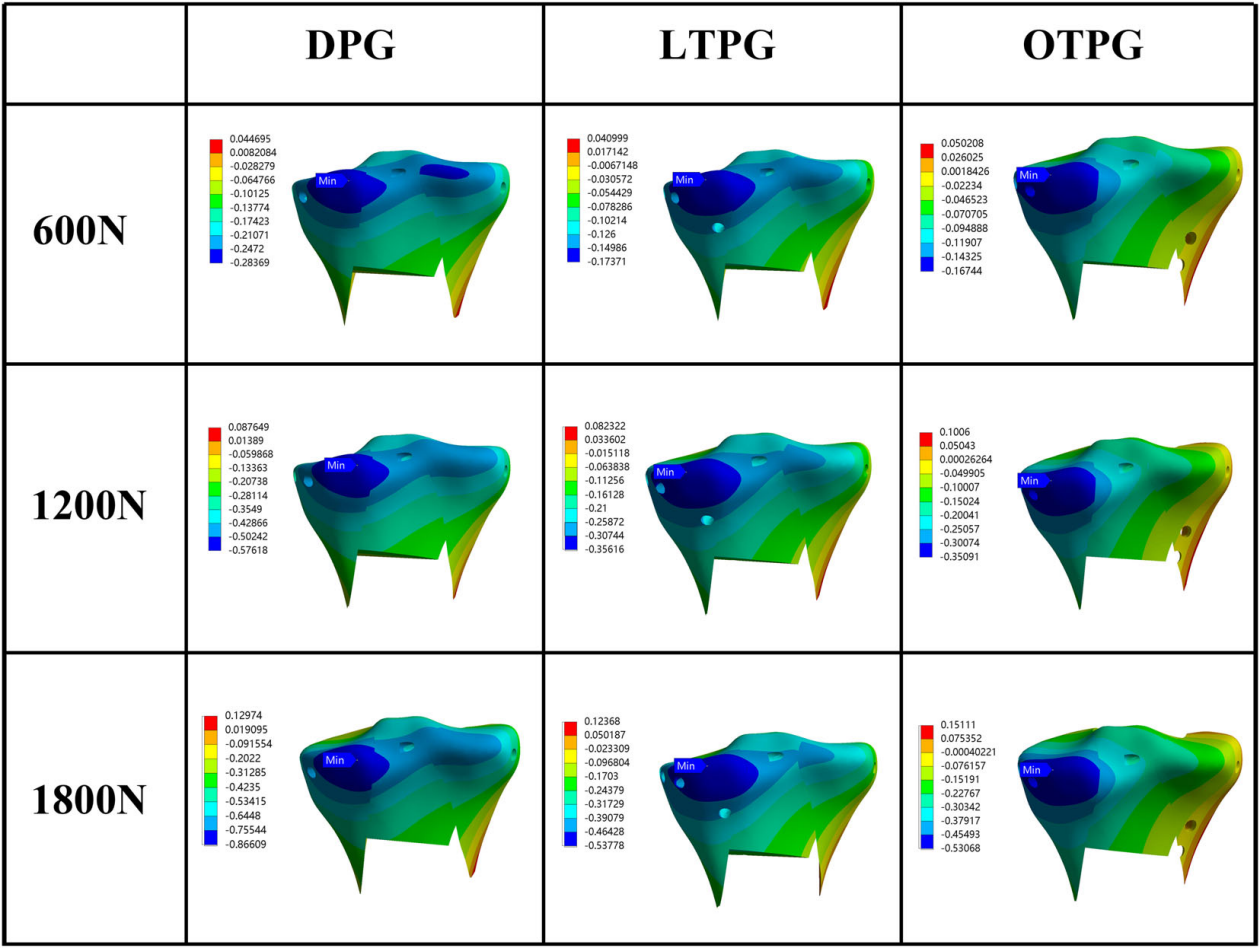
The nephogram of the maximum axial deformation and distribution of the cancellous posterior column (MPCC) under different loading conditions (unit: mm).

**FIGURE 4:**
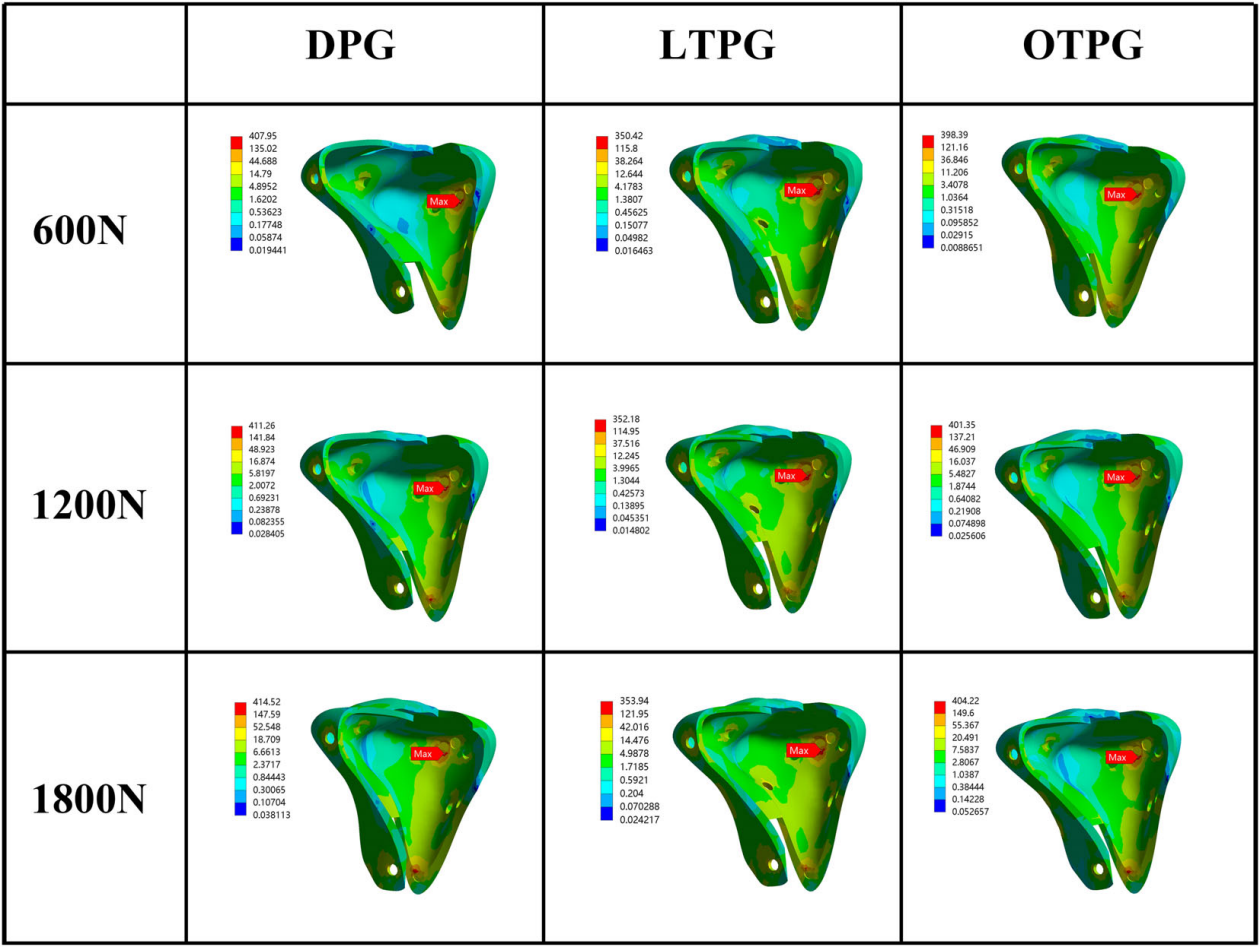
The nephogram of the maximum von Mises stress and distribution of the cortical posterior column (MPC‐VMS) under different loading conditions (unit: MPa).

The MPCC and the MPC‐VMS served as the main parameters of assessing the stability of the posterior column. In all loading scenarios, the MPCC of all models was located along the cancellous posteromedial column's slope, and as compared with DPG, the collapse area (Figure 3, blue region) narrowed down in LTPG and OTPG. In the same loading condition, both the posteromedial and the posterolateral column sank more in LTPG compared with OTPG. Under the force load of 600N, the MPCC was 0.28, 0.17, 0.17 mm in DPG, LTPG, and OTPG, increasing to 0.58, 0.36, 0.35 mm in 1200N, and continuously increasing to 0.87, 0.54, 0.53 mm in 1800N, respectively (Figure 3). The peak stress area (Figure 4, red, orange, and yellow region) grew in size as compared with DPG in both LTPG and OTPG; however, it was obviously smaller in LTPG. Under the force load of 600N, the MPC‐VMS was 408, 350, 398 MPa in DPG, LTPG, OTPG, increasing to 411, 352, 401 MPa in 1200N, and increasing to 414, 354, 404 MPa in 1800N, respectively (Figure 4). The MPC‐VMS of LTPG reduced by 14% and 2% of OTPG when compared with DPG. The comparative results are shown in Figure 7B,D.

### The Overall Resistant Property of Plates and Screws

**FIGURE 5:**
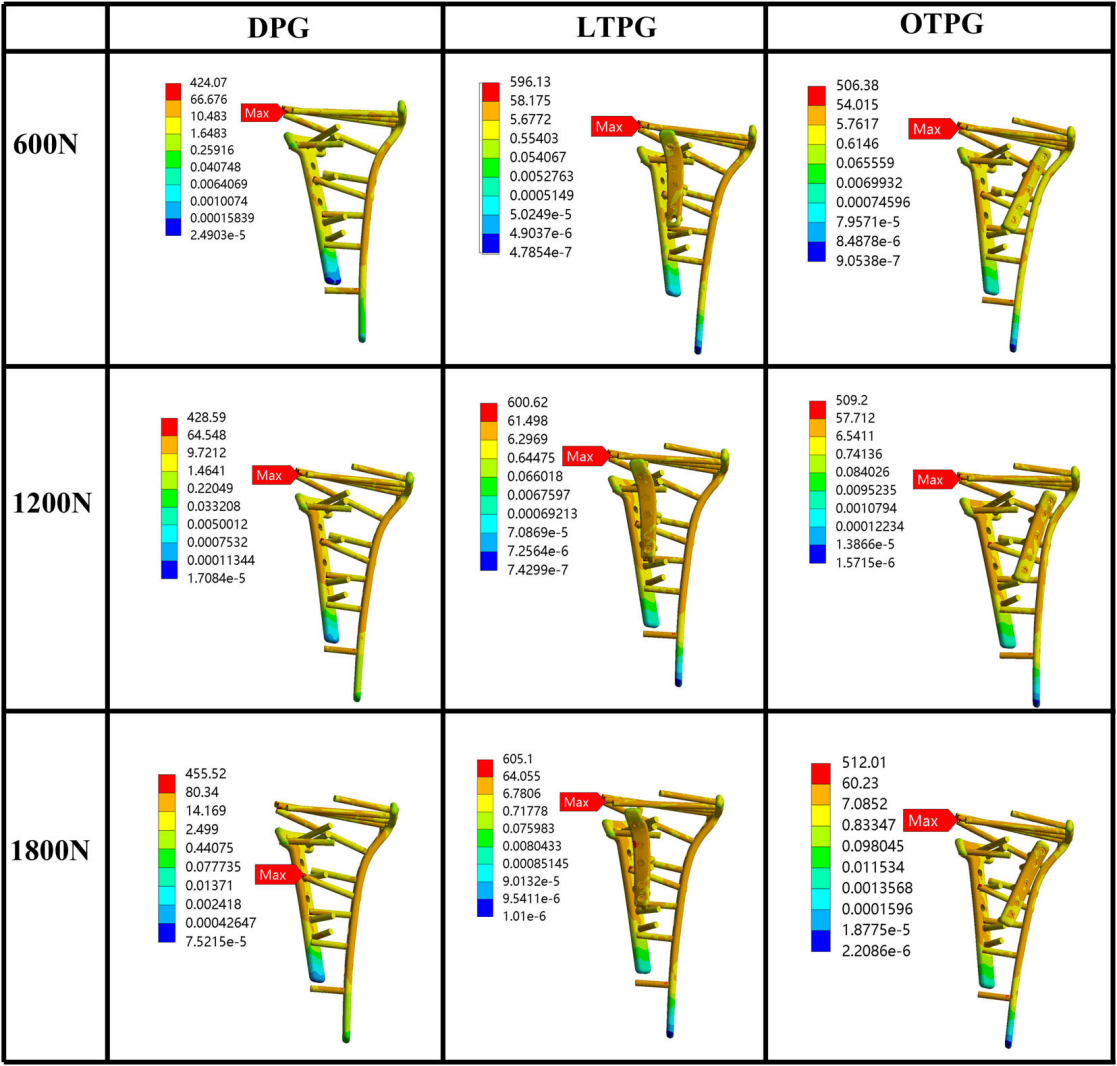
The nephogram of the maximum von Mises stress and distribution in the plates and screws (MPS‐VMS) of three FE models under different loading conditions (unit: MPa).

The MPS‐VMS was located at the distal end of the second bi‐cortical raft screw. The maximum stress area did not alter after the posterior plate was applied. However, the peak stress concentration area (Figure 5, orange region) in raft screws expanded. Otherwise, the peak stress area decreased when the posterior plate was at the oblique position rather than the longitudinal position (Figure 5). Under the force load of 600N, the MPS‐VMS was 427, 596, 506 MPa in DPG, LTPG, and OTPG, slightly increasing to 428, 600, 509 MPa in 1200N, and progressively increasing to 455, 605, 512 MPa in 1800N.

### The Stability of the Combined Models

**FIGURE 6:**
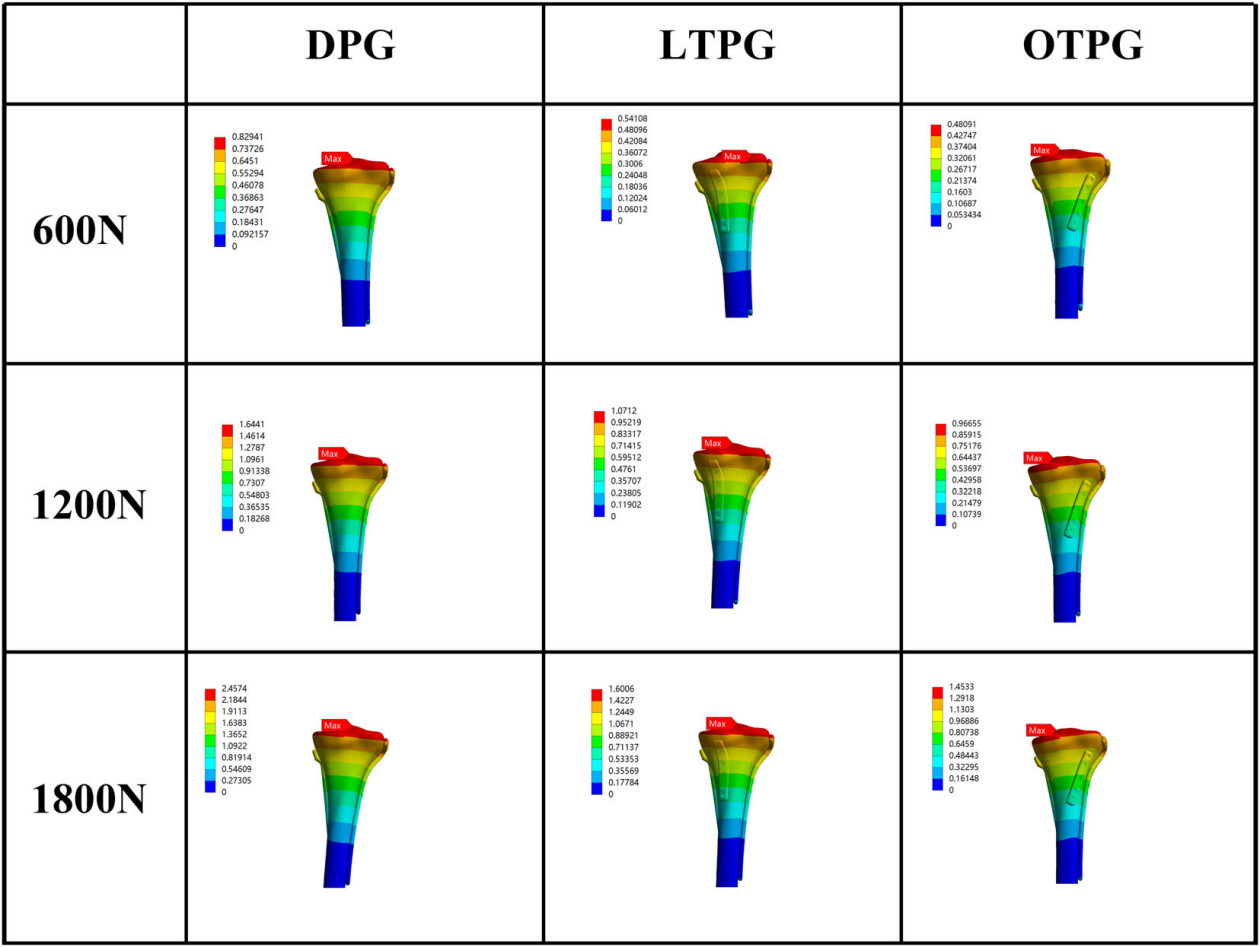
The nephogram of the maximum deformation and distribution of the overall FE models (MTD) under different loading conditions (unit: mm).

**FIGURE 7:**
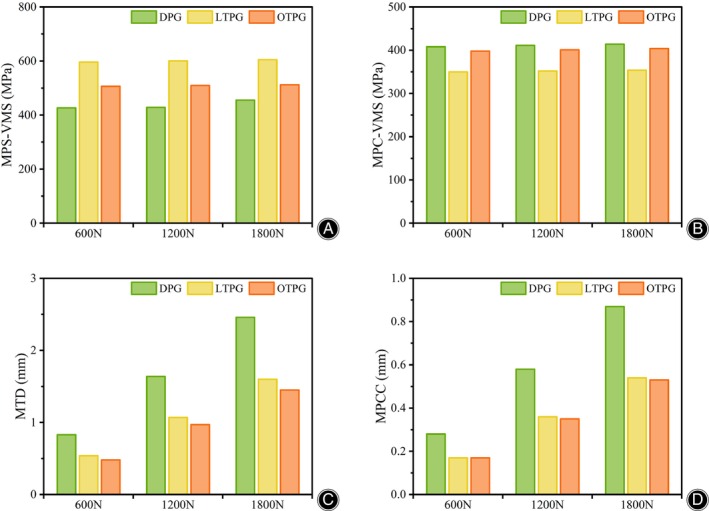
The histogram comparison of four evaluation parameters under different loading conditions. (A) The maximum von Mises stress of the plates and the screws (MPS‐VMS). (B) The maximum von Mises stress of the posterior column (MPC‐VMS). (C) The maximum displacement of the overall FE models (MTD). (D) The maximum displacement of the posterior column fragment (MPCC).

The MTD increased as the load force increased, as shown in Figure 6. The MTD was 0.83, 0.54, and 0.48 mm in DPG, LTPG, and OTPG under the 600N force load. It increased to 1.64, 1.07, 0.97 mm in 1200N and 2.46, 1.60, 1.45 mm in 1800N. The MTD decreased by 35% in LTPG and 42% in OTPG in 600N, 35% in LTPG, and 41% in OTPG in 1200N and 1800N as compared with DPG. The comparative results are shown in Figure 7C.

### Clinical Research Verification

**FIGURE 8:**
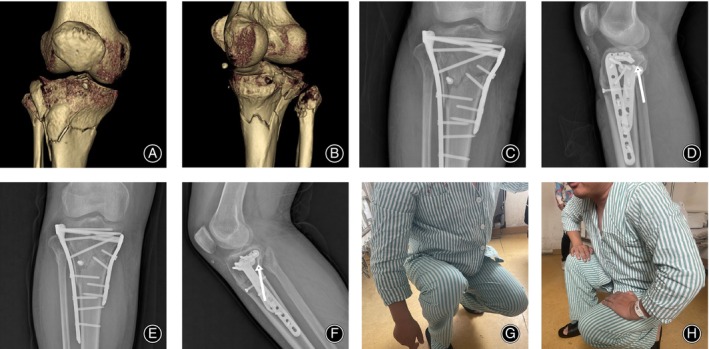
Negative indicator associated with poor functional outcomes by a three‐column fractured case treated by the dual‐plate method. (A, B) The preoperative three‐dimensional CT image revealed that the three columns were all involved. (C, D) Postoperative X‐rays showed that the dual‐plate method could not maintain the anatomical reduction and the arrow signified a backward posterior tibial slope which could not be restored anatomically even though an anteroposterior screw has been applied. (E, F) The backward posterior slope was obvious at the finial follow‐up (14 months). (G, H) The limited knee flexion at the final follow‐up and the patient went for the hardware removal owing to frequent pain and muscle strength weakness, especially during bending.

**FIGURE 9:**
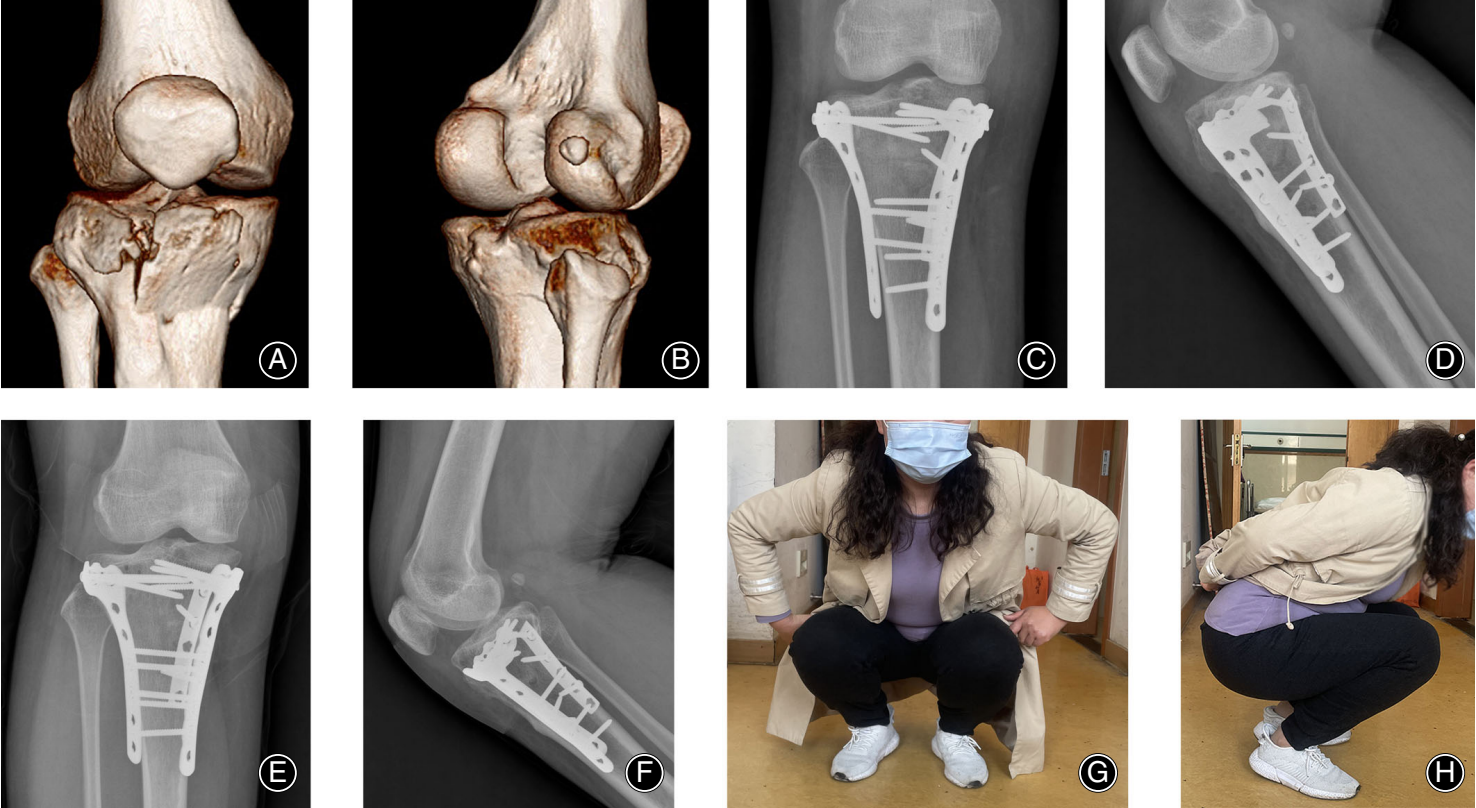
A 41‐year‐old female underwent a motor vehicle accident classified as a hyperextension three‐column fracture and was treated by the triple‐plate method. (A, B) The preoperative three‐dimensional CT image demonstrated that the posterior column was isolated and broken. (C, D) One‐month postoperative X‐rays showed the anatomical reduction was realized by the triple‐plate method. (E, F) A satisfactory anatomical alignment at 6 months. (G, H) Excellent functional knee flexion at final follow‐up (16 months).

The average follow‐up period was 16.2 months in the triple‐plate group and 15.1 months in the dual‐plate group (*p* > 0.05). The mean operation time was 115.6 min in the triple‐plate group and 100.5 min in the dual‐plate group (*p* < 0.05). The mean intraoperative blood loss was 287.0 mL in the triple‐plate group and 206.6 mL in the dual‐plate group (*p* < 0.05). The mean full‐weight bearing period was 14.5 weeks in the triple‐plate group and 16.2 weeks in the dual‐plate group (*p* < 0.05). The mean HSS score was 85.0 in the triple‐plate group and 77.5 in the dual‐plate group (*p* < 0.05). The mean Rasmussen score was 24.1 in the triple‐plate group and 21.6 in the dual‐plate group (*p* < 0.05). The HSS score was evaluated as excellent (for a score above 85), good (70–84), moderate (60–69), poor (below 59), and the Rasmussen score was evaluated as excellent (for a score above 27), good (20–26), moderate (10–19), poor (below 9). In our case series, two cases from the dual‐plate group were assessed as moderate in both functional assessment systems, and the rest were classified as excellent or good (HSS score, triple‐plate: 9 as excellent and 5 as good; dual‐plate: 8 as excellent and 11 as good. Rasmussen score, triple‐plate: 12 as excellent and 2 as good; dual‐plate: 7 as excellent and 12 as good); none of the cases had a poor outcome. One superficial incision infection was found in the triple‐plate group and healed with intravenous antibiotics. No case in either group showed other postoperative complications (Table 3). Figure 8 presented the negative indicators that lead to poor outcomes might be produced if the anatomical reduction of the posterior column could not be realized. Notably, two cases of reduction loss were found in dual‐plate group (9.5%) and no case was found in triple‐plate group (0%). Figure 9 exhibited the anatomical reduction of the posterior column could be maintained well by a posterior implant and it contributed to a satisfactory functional outcome.

**TABLE 3:** Evaluation of the clinical outcomes (mean ± SD)

Indicators	Triple‐plate group (14)	Dual‐plate group (21)	*t*	*p*‐value
Follow‐up period (months)	16.2 ± 4.9	15.1 ± 4.0	0.7	>0.05
Operation time (min)	115.6 ± 7.6	100.5 ± 7.5	5.9	<0.05
Blood loss volume (mL)	287.0 ± 56.9	206.6 ± 57.5	4.1	<0.05
Full‐weight bearing period (weeks)	14.5 ± 2.0	16.2 ± 2.0	2.6	<0.05
Functional outcome				
HSS score	85.0 ± 7.1	77.5 ± 7.1	3.1	<0.05
Rasmussen score	24.1 ± 3.0	21.6 ± 3.1	2.3	<0.05

## Discussion

### Main Findings

In this study, the authors discovered that using a posterior implant in three‐column fractures significantly improves both biomechanics and clinical outcomes. The FE analysis revealed that the posterior plate reduced maximum collapse by at least 38% and overall displacement by at least 35%, effectively preventing secondary displacement. Clinical findings supported these results, with the triple‐plate group showing superior Rasmussen and HSS scores compared with the dual‐plate group, suggesting earlier functional exercise and shorter full‐weight bearing periods. However, despite these benefits, the triple‐plate approach also showed disadvantages like longer surgery time, more blood loss, and a higher risk of superficial incision infection.

### Biomechanical Benefits of Posterior Plate

The significance of the posterior column reduction is substantial. A biomechanical study has reported that the presence of a posterior fracture line reduced fracture construct stiffness by 43% and decreased the failure load by 38% and the maximum displacements were observed at the medial aspect between the tibial plateau and the tibial shaft in the longitudinal direction.^31^ Yan et al. presented a FE analysis of a novel locking buttress plate designed for simultaneous medial and posterior column fractures. Their study compared the new design implant with traditional medial and lateral plates method, verifying that the better biomechanical stability could be obtained when the posterior column had been enhanced.^32^ In our study, the FE analysis was based on a model with fractures in all three columns. Although the posterior plate increased stress on buttress implants and shared the stress concentration area, it remained within clinically acceptable thresholds. The FE results indicated a significant reduction in maximum collapse (by at least 38%) and overall maximum displacement (by at least 35%) with the use of the posterior plate, suggesting its effectiveness in preventing secondary displacement.

### Clinical Benefits of Posterior Plate

Regarding most bicondylar tibial plateau fractures, earlier studies suggested no significant difference in the isolated and dual plates. However, such research did not compare according to the characteristics of the fracture lines and the injury mechanisms. The optimal indicator of using the isolated plate involves positioning screws as perpendicular as possible to the fracture line, and it was also applicable for some multiplane fractures with the fracture lines parallel.^17^ According to the research of the relevant fracture mappings, most of the fracture lines of the bicondylar fractures were not satisfied with this condition. Earlier studies on bicondylar tibial plateau fractures indicated no substantial variance between isolated and dual plates. However, these studies did not account for differences in fracture line characteristics and injury mechanisms. The ideal criterion for utilizing an isolated plate hinges on positioning screws as perpendicular to the fracture line as feasible, which is also relevant for certain multiplane fractures with parallel fracture lines.^17^ Analysis of pertinent fracture mappings reveals that the majority of fracture lines in bicondylar fractures do not meet this criterion.^16^, ^17^ Medial fracture lines often exhibit greater variability compared to lateral lines in complex injury scenarios involving combinations of extension or flexion with varus or valgus forces.^18^ Hence, employing both medial and lateral dual plating is often justifiable for most bicondylar fractures. However, when facing three‐column fractures, where fracture lines typically span at least three distinct orientations, addressing the posterior column with a plate becomes essential. The widespread adoption of the “three‐column” concept has led to various viewpoints advocating for posterior column fixation and corresponding techniques. Sun et al.^33^ reported 13 cases of high‐energy complex tibial plateau fractures treated with a combination of 2.7 mm rim plates for the posterior column and proximal tibial plateau plates for other areas. With an average follow‐up of 4.3 years, patients achieved a mean HSS knee score of 96.2 with no complaints of knee pain. Adequate buttressing strength and space can more effectively support the “bare area” of the posterior column.^34^ Franulic et al.^35^ A recent cadaveric biomechanical study on bicondylar fractures highlighted that medial and lateral locking compression plates might delay full weight‐bearing due to insufficient strength when lacking support from bony union, especially in cases where the posterior column fracture is not addressed.^36^ These findings partly explain why cases treated with the dual‐plate method in our study showed lower functional outcomes, likely due to inadequate posterior anatomical reduction and early rehabilitation. Hence, the concept of column‐specific fixation is crucial in managing complex tibial plateau fractures. However, it's important to acknowledge drawbacks to the triple‐plate approach, including longer operation times, increased blood loss, and higher risks of superficial incision infection. These factors should be carefully considered in surgical planning.

### Exploration of Placement of Posterior Plate

The FE analysis results showed that LTPG and OTPG had comparable effects in preventing posterior column collapse. Although a greater MPC‐VMS was found in OTPG, the MPCC of OTPG was inferior, which was reasonable due to the different construct distribution in the posterior fragment, additionally, the numerical value of each was extremely close, thus it could be seen that both methods had comparable biomechanical stability in the fractures of both the posteromedial and posterolateral columns involved, but if taking the operative factors into account, OTPG seems more adaptive in reality for its border blocking area. In our clinical experience, the posterior plate played an important role in blocking the fragment which contributed to maintaining the posterior reduction during the surgery, and the posterior column tended to the movement if the posterior blocking effect was deficient. For patients with posterolateral fragments or both posteromedial and posterolateral fragments, the oblique placement proved efficacious and the longitudinal placement appeared more appropriate for the patients with posteromedial columns involved. Moreover, the enhanced posterior column facilitated stronger mechanical support to hold early rehabilitation that was linked to better knee function.

### Limitations and Strengths

In this study, the authors innovatively emphasized the importance of posterior column fixation in three‐column fractures. We discovered that a posterior implant significantly aids in maintaining satisfactory reduction, which directly correlates with improved functional outcomes. This finding supports the concept of column‐specific fixation and is recommended for future application. However, there are some limitations to consider. First, the finite element (FE) experimental results were based on the “bicondylar four‐quadrant fracture” model, a typical example of complex three‐column fractures, but it might not encompass all variations. Second, the boundary and loading settings focused solely on the tibial plateau, omitting other force‐transmitting tissues like muscles and ligaments. Last, due to a limited number of cases, the authors did not differentiate the triple‐plate group into sub‐groups of LTPG and OTPG for further clinical outcome comparison, even though differences in fracture distribution might render such comparisons challenging.

## Conclusion

The FE computerized calculation proved that a supplemental posterior plate could be devoted to setting up a resistant barrier for the posterior column and effectively prevent secondary subsidence and malalignment. In the clinical study, a better knee functional outcome in the patients treated with the column‐specific method was attained. Overall, the supplemental posterior plate is beneficial to realizing a rigid reduction and favorable clinical outcomes in the three‐column fractures.

## Conflict of Interest Statement

All authors declare that they have no competing interests.

## Ethics Statement

This study was approved by the institutional review board of Yangpu Hospital, Tongji University School of Medicine (LL‐2018‐ZRKX‐024). Written informed consent was obtained from all patients.

## Author Contributions

Chen‐dong Liu designed the study. Chen‐dong Liu finished the computerized analysis and the statistical analysis. Chen‐dong Liu and Sun‐jun Hu collected and analyzed the patients' clinical data. Chen‐dong Liu drafted the manuscript. Sun‐jun Hu and Shi‐min Chang modified the final version of the manuscript. Shou‐chao Du, Yong‐qian Chu, and Wen‐feng Xiong contributed to the follow‐up. All authors read and approved the final manuscript.

## Data Availability

The data used and/or analyzed during the current study are available from the corresponding author upon reasonable request.
